# N-Acetylglutamate Synthase Deficiency Due to a Recurrent Sequence Variant in the N-acetylglutamate Synthase Enhancer Region

**DOI:** 10.1038/s41598-018-33457-0

**Published:** 2018-10-18

**Authors:** Monique Williams, Alberto Burlina, Laura Rubert, Giulia Polo, George J. G. Ruijter, Myrthe van den Born, Véronique Rüfenacht, Nantaporn Haskins, Laura J. C. M. van Zutven, Mendel Tuchman, Jasper J. Saris, Johannes Häberle, Ljubica Caldovic

**Affiliations:** 1 Department of Metabolic Diseases, Erasmus Medical Center, Sophia Children’s Hospital, Rotterdam, The Netherlands; 2 Department of Pediatrics, Metabolic Unit, University Hospital, University of Padua, Padua, Italy; 3 Department of Clinical Genetics, Erasmus Medical Center, Rotterdam, The Netherlands; 4 Division of Metabolism and Children’s Research Center, University Children’s Hospital, Zurich, Switzerland; 5 Children’s Research Institute, Children’s National Medical Center, Washington, DC 20010 USA

## Abstract

N-acetylglutamate synthase deficiency (NAGSD, MIM #237310) is an autosomal recessive disorder of the urea cycle that results from absent or decreased production of N-acetylglutamate (NAG) due to either decreased NAGS gene expression or defective NAGS enzyme. NAG is essential for the activity of carbamylphosphate synthetase 1 (CPS1), the first and rate-limiting enzyme of the urea cycle. NAGSD is the only urea cycle disorder that can be treated with a single drug, N-carbamylglutamate (NCG), which can activate CPS1 and completely restore ureagenesis in patients with NAGSD. We describe a novel sequence variant NM_153006.2:c.-3026C > T in the NAGS enhancer that was found in three patients from two families with NAGSD; two patients had hyperammonemia that resolved upon treatment with NCG, while the third patient increased dietary protein intake after initiation of NCG therapy. Two patients were homozygous for the variant while the third patient had the c.-3026C > T variant and a partial uniparental disomy that encompassed the NAGS gene on chromosome 17. The c.-3026C > T sequence variant affects a base pair that is highly conserved in vertebrates; the variant is predicted to be deleterious by several bioinformatics tools. Functional assays in cultured HepG2 cells demonstrated that the c.-3026C > T substitution could result in reduced expression of the NAGS gene. These findings underscore the importance of analyzing NAGS gene regulatory regions when looking for molecular causes of NAGSD.

## Introduction

N-acetylglutamate synthase (NAGS; EC 2.3.1.1) is a urea cycle enzyme that catalyzes formation of N-acetylglutamate (NAG) from glutamate and acetyl coenzyme A^1^. NAG is an essential allosteric activator of carbamylphosphate synthetase 1 (CPS1, EC 6.3.4.16), which catalyzes the initial step of ureagenesis – ATP-dependent synthesis of carbamylphosphate from ammonia and bicarbonate^2^. Primary NAGS deficiency (NAGSD, MIM #237310) results from defects in *NAGS* gene and protein leading to decreased abundance or absence of NAG, decreased or absent CPS1 activity, reduced or absent urea synthesis and hyperammonemia^3,4^. Hyperammonemia is the principal biochemical symptom of NAGSD which manifests clinically with nausea, vomiting, cognitive changes, seizures and, in severe cases, coma and death; other biochemical symptoms include high plasma glutamine and low or absent plasma citrulline^4^. Additionally, NAGSD can arise secondary to defects in organic and fatty acid metabolism^3,4^.

Human *NAGS* gene is located on chromosome 17 within band 17q21.31; *NAGS* spans approximately 8.5 kb and has seven exons that encode a 1605 bp open reading frame, starting at chr17:42,082,032 (GRCh37/hg19), six introns, a promoter, and an enhancer located about 3 kb upstream of the transcription start sites^5–9^. The *NAGS* promoter directs transcription of the *NAGS* gene from multiple transcription start sites located between 20 and 120 bp upstream of the NAGS translation initiation codon^9^. The *NAGS* promoter controls transcription of the *NAGS* gene through binding of specificity protein 1 (Sp1) and cAMP response element binding (CREB) transcription factors^9^. The *NAGS* enhancer binds hepatic nuclear factor 1 (HNF1) and nuclear factor Y (NF-Y) transcription factors and is responsible for liver-specific expression of the *NAGS* gene^9^.

Primary NAGSD is an autosomal recessive disorder that affects approximately 1 in 2,000,000 people^10^. Majority of patients with NAGSD are homozygous for the disease causing *NAGS* allele^11^. Most NAGSD causing sequence variants are within *NAGS* coding region or affect splicing of the *NAGS* mRNA^11–15^; a sequence variant in the *NAGS* enhancer (NM_153006.1:c.-3064 C > A) caused NAGSD by reduced NAGS expression due to decreased binding of HNF1 to its binding site^8^. Quick and accurate diagnosis of NAGSD is essential because it is the only urea cycle disorder that can be completely treated with a single drug, i.e. N-carbamylglutamate (NCG), a chemical analog of NAG that binds to and activates CPS1^16,17^ to completely restore ureagenesis in patients with NAGSD^4,18^. NAGSD can be diagnosed by sequencing of the *NAGS* gene and the diagnosis is supported by the markedly enhanced ureagenesis in response to treatment with NCG^5–8^.

We describe a novel sequence variant in the *NAGS* enhancer that occurred independently in two families whose members had NAGSD, totaling to three independent families with NAGS enhancer variants with apparent clinical consequences. This novel variant is pathogenic because it is associated with recurrent episodes of hyperammonemia (and epilepsy) that were promptly resolved upon treatment with NCG and caused reduced expression in a functional assay consisting of a reporter gene in cultured cells.

## Materials and Methods

All methods were performed in accordance with the relevant guidelines and regulations of the participating institutions. Mutation analysis was conducted with the approval of the Institutional Review Boards of the Erasmus Medical Center of the Sophia Children’s Hospital and the University Hospital of the University of Padua. Informed consent was obtained either from all study subjects or their parents/guardians.

### Subject 1

#### History

The female subject is the only child of non-consanguineous parents. Mother is from Russia while father is Dutch. Pregnancy, delivery and neonatal period were normal. She was breastfed until the age of 3 months. She had a normal development, smiling, first words at 6 months and walking at 18 months. At the age of 2–3 years, she refrained from milk and dairy products, fish and meat.

#### Presentation

A female patient, 3 years and 9 months old, was referred because of encephalopathy (Glasgow coma scale, GCS, 6). Her CT scan indicated diffuse bilateral obliteration of peripheral cerebral spinal fluid, hypodensity of white matter with loss of white-gray demarcation, and diffuse hypodense aspect of the white matter as a result of global cerebral ischemia. Four days prior, she had been restless, drowsy/lethargic, and repeating incomprehensible sentences. She seemed tired, but would get up in the night. With increasing loss of consciousness, she was admitted to the hospital. An EEG was suspicious for focal epilepsy. She was treated with diazepam and valproate. Brain MRI, taken after treatment, was normal. Laboratory investigations revealed compensated metabolic acidosis (pH 7.50, pCO2 2.4 kPA, HCO_3_ 18.6 mmol/L, BE −7), elevated lactate 4.0 mmol/L (ref. 1.0–1.8) and ammonia (314 and 207 µmol/L, ref. <50). Amino acids in plasma (prior to treatment) showed increased alanine 1043 µmol/L (ref. 176–480), normal glutamine 482 µmol/L (ref. 400–720), and decreased citrulline 10 µmol/L (ref. 17–50) and arginine 17 µmol/L (ref. 32–128); isoleucine, leucine and valine were also decreased. Urinary orotic acid was normal as was blood acylcarnitine profile. She was started on a high caloric drip, without protein but did not improve clinically and was therefore transferred to the Pediatric Intensive Care Unit (PICU). The patient suffered a similar period with abnormal behavior a month prior to this event, but the cause of her strange behavior remained unclear.

#### Clinical follow up

After admission at PICU, patient was temporarily ventilated because of the low GCS. In addition to hypercaloric intake (need for insulin due to hyperglycemia), she was started on i.v. arginine (250 mg/kg/d loading dose, then same dose as continuous infusion over 24 h, plus nitrogen scavengers sodium benzoate and sodium phenylacetate (250 mg/kg as initial loading dose followed by maintenance dose at 250 mg/kg/d). Ammonia levels decreased from maximum 462 µmol/L to 103 µmol/L, but increased again after 3 days to 194 µmol/L. Therefore, nitrogen scavengers were increased to 400 mg/kg/d and NCG started simultaneously (5 × 200 mg/d). With this treatment and after reintroduction of protein at final dose of 1 g/kg/d, ammonia levels normalized and remained normal.

Based on initial results, ornithine transcarbamylase (OTC) deficiency was suspected and NCG treatment was stopped. Two weeks later, another episode of hyperammonemia occurred which resolved immediately after reintroduction of NCG as the only treatment. This fast response to NCG together with the self-imposed protein restriction suggested NAGSD.

Routine DNA analysis of *NAGS* and *CPS1* coding exons, including the flanking intronic regions, did not reveal any sequence variants. Liver enzyme activity analyses were all normal: NAGS 94 µIE/mg protein (ref. >34), NAGS after arginine stimulation 197 µIE/mg protein (ref. >144), CPS1 26.7 mIE/mg protein (ref. >12), and OTC 489 mIE/mg protein (ref. >160).

**Figure 1:**
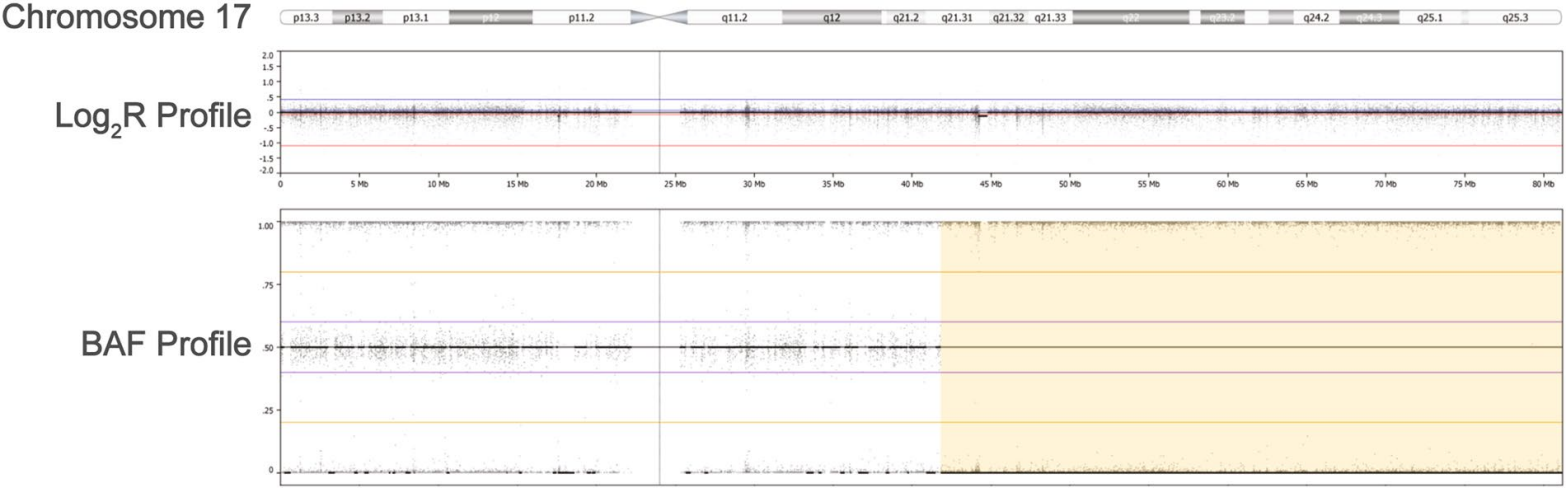
Patient 1 SNP Array results, Illumina HCS850 hg19. Top – Chromosome 17 ideogram, NAGS is located in band 17q21.31, starting at g.42,082,032(GRCh37/hg19). Middle – copy number Log_2_R profile of individual probes (range −2 to 2). Bottom – Heterozygosity plot of polymorphic probes (SNPs) using individual BAF values (range 0 to 1). The patients ROH (yellow shaded segment) as called by software starts at g.41,823,402, and spans 39 Mb and 14 k probes.

A novel homozygous c.-3026C > T variant in the *NAGS* enhancer region was identified (Figure S1 and Supplementary Sequencing Results). The mother tested heterozygous for this variant, while the father did not carry the mutation. Parallel genetic testing of patient’s DNA using SNP array revealed one single, unusually large, 39 Mb, region of homozygosity (ROH). This region contains most of 17q21.31, including the *NAGS* gene, and extends until the telomere. These results are consistent with a monosomic rescue resulting in a partial uniparental disomy (UPD) of chromosome 17 (Fig. 1). A common SNP rs151305517 (MAF 21%), that was detected in DNA from peripheral blood of the father, was absent from the DNA of patient 1 and her mother, thus strengthening the diagnosis of maternal UPD.

### Subject 2

#### History

Subject 2 is a female, who was born at term after an uncomplicated pregnancy. Her physical examination was normal and she was discharged home at 4 days old. In the first years of life, she had normal physical and neurological development. When she was 6 years old, she presented with an episode of fever and vomiting with slow speech, anxiety, confusion, disorientation and altered consciousness requiring hospitalization.

Review of the past medical history was notable for poor feeding and problematic eating habits with rejection of meat and fish. Her weight and height were at the 3^rd^ percentile. Her medical history also revealed that during episodes of fever, she suffered similar episodes of vomiting and slow speech attributed to infections. The mother described increasing tiredness for some days prior to the episode.

#### Presentation

At admission, patient’s neurological examination demonstrated no focal signs and no gait disturbances. Both EEG and brain CT were normal. Laboratory investigation including blood cell count, electrolytes, glucose, liver function, renal function, lactate, blood gas analysis and urinary ketones were all normal. No signs of intoxications: blood alcohol, barbiturate, diphenylhydantoin, digoxin and benzodiazepines were not found. A portosystemic shunt was ruled out with abdominal ultrasound and MRI analysis.

Her blood ammonia level was 251 µmol/L (reference range 0–35) which remained elevated despite continuous i.v. glucose fluids. Due to persistent hyperammonemia, treatment with sodium benzoate was initiated. A complete metabolic work-up including the plasma amino acid profile showed high glutamine (895 µmol/L), alanine (719 µmol/L) while urinary organic acids and blood acylcarnitine profile were normal. No orotic acid was detected in the urine collected during acute hyperammonemic episode. Subsequently, an allopurinol loading test was performed showing neither orotic acid nor orotidine. Screening of the gene panel for UCD, which includes *CPS1*, *OTC*, *ASS1* and *ASL* coding exons did not reveal any pathogenic sequence variants.

Three months after this episode, she presented again with a similar psychotic picture that required hospitalization. Metabolic investigations revealed hyperammonemia (236 µmol/L), elevated glutamine in plasma (855 µmol/L) and in the CSF (2243 µmol/L; ref. 390–824). These data, consistent with a proximal urea cycle defect, including NAGSD, suggested to change therapy from sodium benzoate to NCG at an initial dose of 100 mg/kg/d. Shortly after this change, ammonia normalized and the child regained her normal activity.

#### Clinical follow up

Treatment with NCG was continued at 50 mg/kg/d and the patient has shown no further clinical manifestation even during fever episodes, her appetite improved, including tolerance to natural protein foods and her protein intake could be increased to 1.8 g/kg/d. Patient’s weight and height reached the 50^th^ percentile and the neurological and developmental assessments were normal. Despite the negative results for *NAGS* mutation(s) in the coding region and splice sites we decided to continue the therapy with NCG. After mutation analysis of patient 1 revealed the NM_153006.2:c.-3026C > T variant in the *NAGS* enhancer we revisited the previous sequencing results in this patient and found that she is also homozygous for the c.-3026C > T sequence variant (Figure S1 and Supplementary Sequencing Results). Both parents are heterozygous carriers of the variant, which confirms recessive inheritance and excludes the possibility of a small deletion and/or allele dropout.

### Subject 3

#### History

Subject 3 is the dizygotic twin sister of patient 2. She presented with poor appetite and avoidance of foods with high protein content. Her weight and height were at 5^th^ percentile. Unlike her sister, patient 3 never had the behavioural problems during fever episodes although her mother noticed irritability for few days after the fever subsided.

#### Clinical follow up

After patient’s sister was diagnosed with NAGSD, a metabolic work-up was performed including direct analysis of the *NAGS* enhancer revealing the same homozygous c.-3026C > T change as in patient 2 (Figure S1 and Supplementary Sequencing Results). Pre-prandial plasma ammonia was normal but the postprandial plasma ammonia rose to 85 µmol/L while plasma glutamine increased to 1109 µmol/L. She was put on NCG (50 mg/kg/d) with normalization of biochemical parameters and an unexpected increased appetite for high protein foods.

### Mutation Analysis of the NAGS gene

*NAGS* mutation analysis using DNA from peripheral blood cells was performed as described previously^19^. Sequencing of the *NAGS* enhancer and promoter^8^ was subsequently included in the mutation analysis of the *NAGS* gene.

SNP array analysis of DNA from patient 1 was performed with Illumina Gene Chip 850 in a standard clinical laboratory procedure. Illumina GenomeStudio and Biodiscovery Nexus SE v7.5 were used for genotyping, determination of copy number imbalance, and ROH analysis.

### Bioinformatic Analysis of the NAGS Regulatory Region

Sequence of the *NAGS* upstream regulatory region (chr17:42,0798,560-42,082,100 of the GRCh37/hg19 human genome assembly) and the conservation scores for each position were downloaded from the UCSC Genome Browser (https://genome.ucsc.edu/). Sequences of *NAGS* enhancers from 30 mammals (Table S1) were downloaded from the UCSC Genome Browser and aligned using Clustal Omega^20^. LOGO representation of the multiple sequence alignment was generated using WebLOGO 3^21^.

### Plasmid Construction and Expression Studies

Expression plasmid p4.23hE_Mut, which harbors *NAGS* enhancer mutation found in patients was generated with mutagenic primers 5′- AGAGGGCCATGTCCCCAGGCAGCC-3′ and 5′-GGCTGCCTGGGGACATGGCCCTCT-3′, and p4.23hE plasmid as template^8^ using Quick Change Lightning Mutagenesis kit (Agilent Technologies) according to manufacturer’s instructions. Presence of the enhancer mutation was confirmed by DNA sequencing of the resulting plasmid.

Human HepG2 hepatoma cells (ATCC) were cultured in complete EMEM medium (ATCC) supplemented with 10% fetal bovine serum (ATCC) and 5% penicillin/streptomycin (Life Technologies); cells were cultured at 37 °C in atmosphere containing 5% CO_2_. Cells were plated in the 24-well plates at 1.5 × 10^5^ cells/well; 24 hr. later cultured cells reached 90–95% confluency. Cells were transfected with Lipofectamine 3000 reagent (Life Technologies) mixed with 0.5 µg of plasmid DNA. The ratio of experimental plasmids, which express firefly luciferase *luc2*, and pGL4.74, which expresses *Renilla reniformis* luciferase and was used as a transfection efficiency control, was 1000:1.

Cells were harvested 24 hr. after transfection and used for reporter gene assay with the Dual Luciferase Reporter Assay System (Promega) and Berthold Centro 960 luminometer according to the manufacturer’s instructions. Firefly luciferase activities were normalized to activity of *Renilla* luciferase to correct for differences in transfection efficiency, and to firefly luciferase activity from the p4.23hE plasmid harboring wild-type *NAGS* enhancer. Results are average of three independent experiments, each carried out in triplicate. Values were expressed and mean ± SEM and analyzed using Student t-test.

## Results

### Human NAGS Gene Regulatory Region

**Figure 2:**
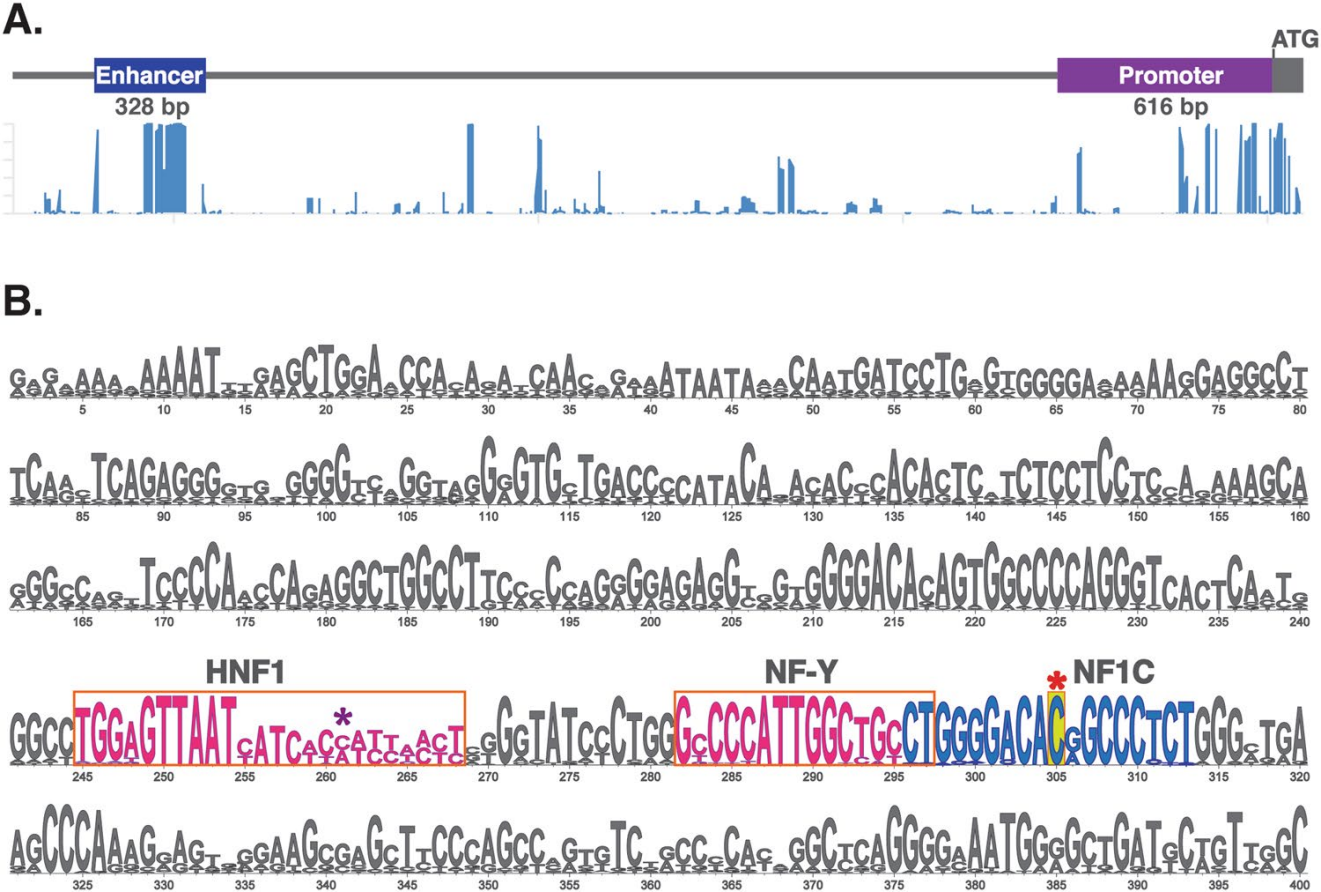
Upstream regulatory region of the human *NAGS* gene. (**A**) Map and conservation of the *NAGS* regulatory region. The *NAGS* promoter and enhancer span respective regions chr17:42,081,416–42,082,031 and chr17:42,078,768–42,079,095 of the GRCh37/hg19 human genome build. (**B**) LOGO representation of the multiple sequence alignment of NAGS enhancers from 30 mammals. The height of each letter corresponds to its conservation. HNF1 and NF-Y binding sites are shown in magenta. The NF1C binding site is shown in blue. Mutated base pair is highlighted in yellow and indicated with red asterisk (*). Magenta asterisk indicates site that was mutated in patient with NAGSD^8^.

Human *NAGS* gene regulatory region encompasses approximately 4 kb; it harbors several regions of high conservation in mammalian genomes that cluster in the *NAGS* promoter, immediately upstream of *NAGS* exon 1, and in the *NAGS* enhancer, which is located about 3 kb upstream of the NAGS translation initiation codon (Fig. 2A). Human *NAGS* enhancer is approximately 300 bp long and binds transcription factors HNF1 and NF-Y that are important for NAGS expression in the liver (Fig. 1B and^8,9^). ENCODE project revealed that human *NAGS* enhancer also binds transcription factor NF1C in HepG2 cells (Fig. 2B and^22^).

### Identification of Disease Causing Sequence Variant in the NAGS Enhancer

All three patients in this study presented with hyperammonemia and a metabolic profile suggestive for a proximal urea cycle disorder. Treatment with NCG led to normalization of plasma ammonia in patients 1 and 2 while withdrawal of this treatment resulted in recurrence of hyperammonemia in both patients. The third patient in this study markedly increased dietary protein intake after initiation of treatment with NCG. Therefore, genetic investigations were expanded beyond the routine sequencing of the coding *NAGS* exons to include the *NAGS* gene regulatory regions.

Sequencing of the *NAGS* regulatory region in all three patients revealed a C to T change at position chr17: 42,079,006 (GRCh37/hg19 human genome assembly), which is 3026 bp upstream of the *NAGS* translation initiation codon, within intron1 of PYY, and within the Nuclear Factor IC (NFIC) transcription factor binding site (Fig. 2B). This sequence variant has not been reported in dbSNP147 and 1000 Genomes project databases; this region has not been released for query in the Genome Aggregation Database (gnomAD, version2.0)^23^. Estimates of evolutionary conservation and computational mutation effect prediction tools were used to determine whether c.-3026C > T sequence variant could be a cause of NAGSD. The genomic evolutionary rate profiling (GERP)^24,25^ and Phylo-P^26^ scores for the base pair affected by the c.-3026C > T variant are 4.51 (on the scale of −12.36 to 6.81) and 2.749 (on the scale of −20 to 7.532), respectively, indicating evolutionary constraint at this position while the PhastCons score^26^ of 0.999 (on the scale of 0–1) indicates that the c.-3026C is part of a conserved element. The CADD (Combined Annotation-Dependent Depletion) scaled C-score, which includes the previous scores in a weighted model, for the c.-3026C > T is 19.36 indicating that this variant is among 1–2% of the most deleterious variants in the human genome^27^, and MutationTaster2^28^ classified this variant as disease causing. Additionally, c.-3026C > T variant was classified as deleterious by each of the five variant prediction tools (CADD, DANN, FATHMM, FunSeq2 and GWAVA) that are combined into PredictSNP2 platform^29^. Because computational prediction of the effects of sequence variants are not always accurate we carried out functional testing in cultured cells.

### Expression Studies

**Figure 3:**
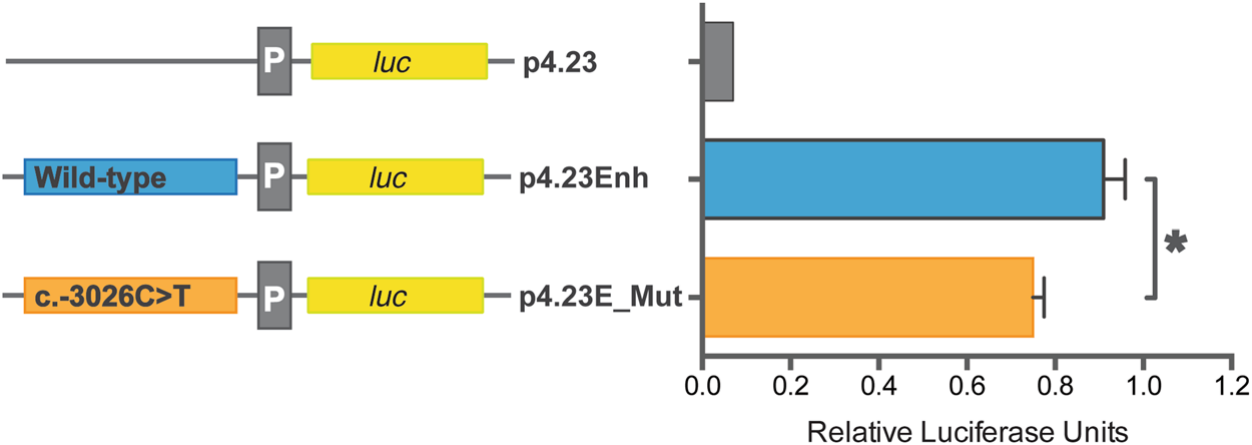
Effect of the c.-3026C > T sequence variant on gene expression. Luciferase expression is significantly different between wild-type and c.-3026C > T enhancers. An asterisk (*) indicates *P* < 0.05.

Reporter gene assays in cultured HepG2 cells were used to test whether the c.-3026C > T sequence variant could be responsible for decreased expression of the *NAGS* gene and NAGSD in three patients. The C to T change corresponding to the c.-3026C > T variant was engineered into p4.23Enh construct^8^ and resulting construct p4.23hE_Mut was transfected into HepG2 cells. The p4.32Enh construct, harboring wild-type *NAGS* enhancer, was used as a positive control and p4.23, which lacks *NAGS* enhancer, was a negative control. The relative firefly luciferase activity was approximately 20% lower in HepG2 cells transfected with the p4.23hE-Mut construct than in cells transfected with the construct harboring wild-type *NAGS* enhancer (Fig. 3). This suggests that the c.-3026C > T *NAGS* sequence variant could cause reduction of *NAGS* gene expression and NAGSD.

## Discussion

Herein we describe a recurrent pathogenic sequence variant c.-3062C > T that resides in the *NAGS* enhancer region and was found in three patients with. Two of the patients from two different families presented with hyperammonemia that resolved upon treatment with NCG; the third patient is a twin sister of one of the patients that presented with hyperammonemia. In all three patients the c.-3026C > T sequence variant, located within *NAGS* enhancer and 3026 bp upstream of the NAGS translation initiation site^8,9^, was the only sequence variant that could explain their disease. In addition to the c.-3026C > T sequence variant, patient 1 also had an extensive ROH that encompasses the *NAGS* gene on chromosome 17, which is consistent with chromosomal rearrangement resulting in partial UPD of chromosome 17. This case highlights the importance of combining metabolic screening, SNP-array, and mutation analysis of DNA in diagnosing NAGSD. Patients 2 and 3 are dizygotic twins and each is homozygous for the c.-3026C > T variant. Unlike patient 1, who had severe neurological symptoms associated with hyperammonemia, patients 2 and 3 did not have seizures or abnormal brain MRI. Both sisters presented with altered behavior associated with febrile episodes, poor feeding, and avoidance of high protein foods prior to NAGSD diagnosis and treatment with NCG. All of these symptoms resolved after treatment with NCG was initiated. This underscores the importance of the response of patient’s hyperammonemia to NCG treatment in diagnosing NAGSD.

The c.-3026C > T variant maps in the vicinity of the NF-Y transcription factor binding site^9^ and within Nuclear Factor IC (NFIC) binding site, which has been identified using chromatin immunoprecipitation followed by sequencing carried out as part of the ENCODE project^30,31^. Although NFIC transcription factor is a DNA-binding protein that interacts with transcription machinery^32^, the presence of its binding site within an enhancer is not unusual as many enhancers have been shown to interact with transcription machinery when regulating expression of their target genes^33^. The high conservation scores of the base pair affected by the c.-3026C > T variant indicate that it resides in a conserved element, and suggests evolutionary constraint in vertebrates and importance for the *NAGS* enhancer function. This is reflected in computational predictions that c.-3026C > T variant is deleterious (PredictSNP2) and disease causing (MutationTaster2). Because computational predictions of the functional effects of sequence variants do not always agree with the expert opinion^34^, we carried out functional testing with reporter gene constructs, which revealed that the c.-3026C > T variant could affect expression of the *NAGS* gene. Taken together clinical, biochemical and molecular evidence strongly suggest that the c.-3026C > T variant is pathogenic and caused NAGSD in three patients experiencing physiological stress. As a consequence, and including the previous single case, we recommend adding sequencing of the *NAGS* enhancer region both in direct *NAGS* gene analysis using Sanger sequencing and when next generation approaches are performed.

## Electronic Supplementary Material

FigureS1 and TableS1

Dataset 1
