# Supplementary material for: N-Acetylglutamate Synthase Deficiency Due to a Recurrent Sequence Variant in the N-acetylglutamate Synthase Enhancer Region
Source: Sci Rep. 2018 Oct 18;8:15436. doi: 10.1038/s41598-018-33457-0 (PMC6194121; doi:10.1038/s41598-018-33457-0)
Supplement: Supplementary file 1 — FigureS1 and TableS1 [file 41598_2018_33457_MOESM1_ESM.pdf]

N-Acetylglutamate Synthase Deficiency Due to a Recurrent Sequence Variant in the N-acetylglutamate Synthase Enhancer Region

Monique Williams[1†], Alberto Burlina[2†], Laura Rubert[2], Giulia Polo[2], George J.G. Ruijter[3], Myrthe van den Born[3], Véronique Rüfenacht[4], Nantaporn Haskins[5], Laura J.C.M. van Zutven[3], Mendel Tuchman[5], Jasper J. Saris[3], Johannes Häberle[4*], Ljubica Caldovic[5*]

[1]Dept. of Metabolic Diseases, Erasmus Medical Center, Sophia Children's Hospital, Rotterdam, The Netherlands, [2]Department of Pediatrics, Metabolic Unit, University Hospital, University of Padua, Padua, Italy, [3]Department of Clinical Genetics, Erasmus Medical Center, Rotterdam, The Netherlands, [4]Division of Metabolism and Children's Research Center, University Children's Hospital, Zurich, Switzerland, [5]Children's Research Institute, Children's National Health System, Washington DC, 20010

[†]These authors contributed to this study equally.

*Corresponding Authors: Ljubica Caldovic, Ph.D.
Children's Research Institute
Children's National Health System
111 Michigan Ave NW
Washington DC, 20010
Tel: (202) 476-5819
Fax: (202) 476-6014
Email: LCaldovic@childrensnational.org

Johannes Häberle
Division of Metabolism and Children's Research Center
University Children's Hospital
Zurich, Switzerland
Tel: +41 44 266 73 42
Fax: +41 44 266 71 67
Email: Johannes.Haeberle@kispi.uzh.ch

Key words: N-acetylglutamate synthase, N-acetylglutamate, urea cycle, ureagenesis, urea cycle disorders, mutation analysis, enhancer

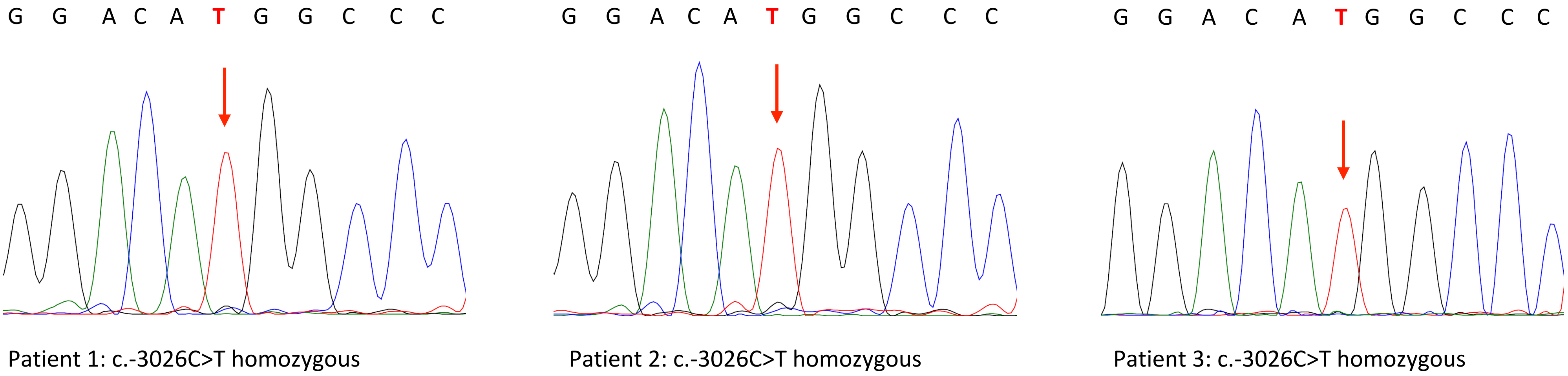

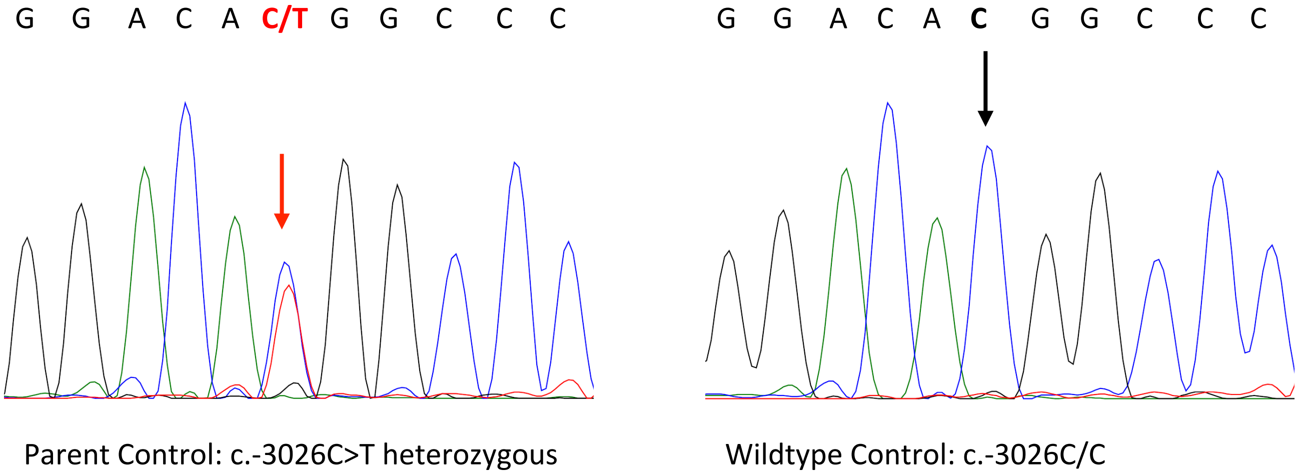

Figure S1. Chromatogram summaries of the sequencing results from the three subjects in this study. Sequencing of the parent's DNA and DNA from an unaffected individual were used as controls.

Table S1. Species names and coordinates of the NAGS enhancer sequences that were used for LOGO sequence alignment.

**Common Name**	**Species name**	**Genomic Region**
Human	*Homo sapiens*	chr17:42078741-42079100
Chimp	*Pan troglodytes*	chr17:13434248-13434607
Gorilla	*Gorilla gorilla*	chr5:40114966-40115325
Orangutan	*Pongo abelii*	chr17:45462296-45462655
Gibbon	*Nomascus leucogenys*	chr19:32756481-32756840
Rhesus	*Macaca mulatta*	chr16:56048102-56048461
Crab-eating macaque	*Macaca fascicularis*	chr16:55750517-55750876
Baboon	*Papio hamadryas*	scaffold10667:11110-11469
Marmoset	*Callithrix jacchus*	chr5:76365618-76365977
Squirrel monkey	*Saimiri boliviensis*	JH378291:2124656-2125015
Chinese hamster	*Cricetulus griseus*	KE383081:1120945-1121304
Mouse	*Mus musculus*	chr11:102142283-102142642
Rat	*Rattus norvegicus*	chr19:44363933-44364286
Rabbit	*Oryctolagus cuniculus*	JH802149:3740620-3740973
Pica	*Ochotona princeps*	chr12:19414709-19415068
Pig	*Sus scrofa*	KB632730:427758-428117
Alpaca	*Vicugna pacos*	ABRN02083350:1-250
Dolphin	*Tursiops truncates*	chr19:44886117-44886476
Cow	*Bos taurus*	chr11:43283195-43283554
Sheep	*Ovis aries*	chr11:19469473-19469832
Horse	*Equus caballus*	JH767765:478006-478365
White rhinoceros	*Ceratotherium simum*	chrE1:44067409-44067768
Cat	*Felis catus*	chr9:19351555-19351914
Dog	*Canis familiaris*	GL192699.1:449853-450212
Panda	*Ailuropoda melanoleuca*	GL429867:451965-452324
Microbat	*Myotis lucifugus*	JH835844:947188-947547
Hedgehog	*Erinaceus europaeus*	scaffold_31:25270628-25270987
Elephant	*Loxodonta Africana*	JH594634:22529983-22530342
Manatee	*Trichechus manatus latirostris*	JH564260:1203523-1203882
Armadillo	*Dasypus novemcinctus*	chr10:89869499-89869858
